# Electrochemical Nano-Imprinting of Trimetallic Dendritic Surface for Ultrasensitive Detection of Cephalexin in Pharmaceutical Formulations

**DOI:** 10.3390/pharmaceutics15030876

**Published:** 2023-03-08

**Authors:** Rohini Kumari, Pranjal Chandra

**Affiliations:** Laboratory of Bio-Physio Sensors and Nanobioengineering, School of Biochemical Engineering, Indian Institute of Technology (BHU), Varanasi 221005, Uttar Pradesh, India; rohinikumari.rs.bce21@itbhu.ac.in

**Keywords:** electrochemical nano-imprinting, cephalexin, trimetallic AuCoCu NDs, surface engineering

## Abstract

Cephalexin (CFX), a first-generation cephalosporin, is used to treat various infectious diseases. Although antibiotics have achieved considerable progress in the eradication of infectious diseases, their incorrect and excessive usage has contributed to various side effects, such as mouth soreness, pregnancy-related pruritus, and gastrointestinal symptoms, including nausea, epigastric discomfort, vomiting, diarrhoea, and haematuria. In addition to this, it also causes antibiotic resistance, one of the most pressing problems in the medical field. The World Health Organization (WHO) claims that cephalosporins are currently the most commonly used drugs for which bacteria have developed resistance. Hence, it is crucial to detect CFX in complex biological matrices in a highly selective and sensitive way. In view of this, a unique trimetallic dendritic nanostructure comprised of cobalt, copper, and gold was electrochemically imprinted on an electrode surface by optimising the electrodeposition variables. The dendritic sensing probe was thoroughly characterised using X-ray photoelectron spectroscopy, scanning electron microscopy, chronoamperometry, electrochemical impedance spectroscopy, and linear sweep voltammetry. The probe displayed superior analytical performance, with a linear dynamic range between 0.05 nM and 10^5^ nM, limit of detection of 0.04 ± 0.01 nM, and response time of 4.5 ± 0.2 s. The dendritic sensing probe displayed minimal response to interfering compounds, such as glucose, acetaminophen, uric acid, aspirin, ascorbic acid, chloramphenicol, and glutamine, which usually occur together in real matrices. In order to check the feasibility of the surface, analysis of a real sample was carried out using the spike and recovery approach in pharmaceutical formulations and milk samples, yielding current recoveries of 93.29–99.77% and 92.66–98.29%, respectively, with RSD < 3.5%. It only took around 30 min to imprint the surface and analyse the CFX molecule, making it a quick and efficient platform for drug analysis in clinical settings.

## 1. Introduction

CFX, an FDA-approved antibiotic, is a first-generation cephalosporin and is used to combat several bacterial infections, such as respiratory and urinary tract infections, gonorrhoea, scarlet fever, middle ear infections, and skin infections [1]. It is also used to treat heart disorders due to its improved oral action [2]. CFX, 7-[(amino-phenylacetyl)amino]-3-methyl-8- oxo-5-thia-1-azabicyclo[4.2.0]oct-2-ene-2-carboxylic acid, is one of the most commonly prescribed oral medications in the world [2]. Though it is highly effective, consuming it too much can be dangerous to people; for instance, prolonged usage of CFX might lead to acute renal failure [3,4]. In addition, there are several other negative side effects attributed to toxicity or overdose, such as pregnancy-related pruritus, mouth soreness, and gastrointestinal symptoms, including nausea, vomiting, epigastric discomfort, diarrhoea, and haematuria [1]. CFX can also result in drug-induced Acute Generalised Exanthematous Pustulosis, Stevens–Johnson syndrome, and toxic epidermal necrolysis [1,5]. Antibiotics have made significant advancements in the eradication of infectious diseases, but their excessive and improper use has led to the rise of antibiotic resistance, one of the most serious issues nowadays [6,7,8]. Infections caused by resistant bacteria ultimately claim the lives of over 25,000 people each year in the European Union alone [9]. According to the WHO, cephalosporins are currently widely used medications for which bacteria have acquired resistance [4]. Therefore, precise and sensitive detection of CFX in various complex matrices and pharmaceutical formulations is essential from a therapeutic point of view. Various techniques for quantifying cephalexin and other cephalosporins have been implemented so far. These techniques are categorised into four groups: chromatographic, spectrophotometric, microbiological, and electrochemical approaches [9]. Despite being the most-often-used detection methods, microbiological techniques have some limitations, the most significant among which is the need for bacterial cell cultures. Cephalosporins have been quantified in various matrices using instrumental methods as well. Instrumental methods include high-performance liquid chromatography (HPLC) combined with mass spectrometry, HPLC coupled with ultraviolet detection (UV), molecularly imprinted solid-phase extraction (SPE) coupled with UV spectrophotometry and HPLC. However, each of the chromatographic techniques mentioned above requires a complex set of operating procedures, skilled staff, and a substantial amount of dangerous chemicals [10]. Several fluorescent sensors have been designed to date for the sensitive and rapid detection of analytes. However, these approaches are expensive, time-consuming, and require a lot of space [11,12]. Further light can interfere with their operation, their stability is limited due to indicator leaching or photobleaching, and the dynamic range of these sensors is narrow [13]. Electrochemical analyses are becoming an appealing option because they are robust, cost-effective, stable, reproducible, offer quick and highly sensitive analytical results, do not require expensive instruments, and can be easily miniaturised [14]. The electro-catalytic and optoelectronic properties of nanomaterials are mainly utilised in sensing systems to improve their sensitivity and signal responsiveness for bio/chemical marker detection [15,16,17,18]. Electrochemical deposition, in comparison with other nanomaterial production methods, is a reliable, fast, and affordable method for synthesising a uniform layer of nanomaterials on a surface at optimum potential. It is a simple and direct method for altering alloy composition to construct distinctive multi-layers of nanodendrites that are challenging to obtain by utilising other approaches [19]. The added benefit of this method is that it reduces the errors in the current signal caused by the inaccurate transfer of material on the surface of electrodes [20]. Metallic nanodendrites (NDs), because of their three-dimensional (3D) morphology, are notably distinctive and remarkable in their architectural design among metallic nanomaterials with unique structural morphologies, such as nanorods, nanoplates, nanoprisms, nanocages, nano-dumbbells, nanostars, etc. [21,22]. They consist of a main stem and several lateral branches, and have a vast surface area and remarkable conductivity [23,24]. Due to the abundance of edges, ridges, and corner atoms, metallic nanodendrites display a large number of catalytically active sites [25]. Trimetallic NDs outperform mono and bimetallic NDs in terms of their physical/chemical stability and the synergistic effects among the three metals [26]. Hence, designing trimetallic NDs could be an effective approach to further enhance the number of catalytic active sites and surface area. So far, there has been no report of a transduction surface comprising a dendritic sensing probe made up of gold, cobalt, and copper metal for sensing any molecule, including CFX.

**Scheme 1:**
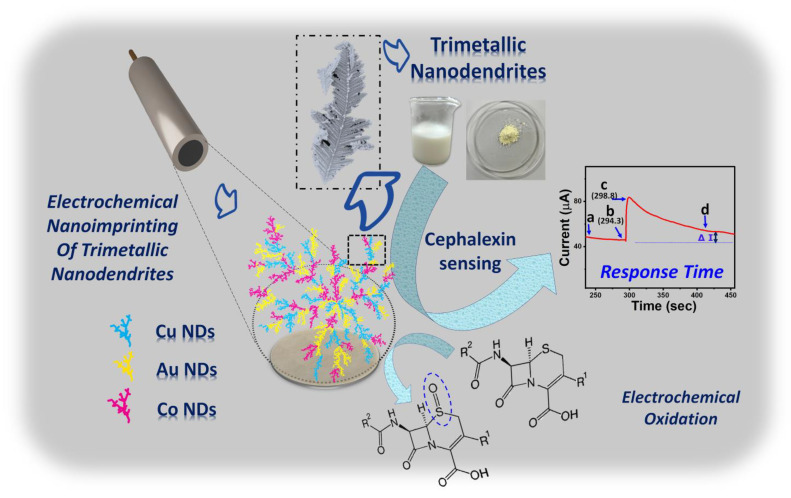
Schematic depicting the nanoimprinting of the trimetallic dendritic sensing probe and its feasibility for the testing of CFX in pharmaceutical formulation and milk sample. The response time curve is showing a chronoamperometric response of the GCE/AuCoCu dendritic sensing probe with a detection time of 4.5 ± 0.2 s; (a) represents the stable current; after injecting the CFX at point (b), a sharp spike in current (c) due to diffusion was recorded between 294.3 and 298.8 s; following that, current saturation occurred (d).

In the current study, a novel trimetallic AuCoCu NDs was electrochemically printed using a controlled electrodeposition process over the surface of a glassy carbon electrode (GCE) with a mirror-like finish. The nanoimprinted surface exhibited improved electrical and catalytic properties and was eventually employed for the direct detection of CFX. To further study the conducting and catalytic behaviour of the sensor surface, electrochemical analyses were carried out using chronoamperometry, linear sweep voltammetry (LSV), and electrochemical impedance spectroscopy (EIS). The analytical potency of the dendritic sensing probe for detection was assessed using the limit of detection (LOD) and linear dynamic range (LDR). Pharmaceutical formulations in the form of capsules and milk samples were examined to analyse the applicability of the imprinted sensing surface for CFX detection. The reproducibility of the sensor over time, as well as the interference effects by coexisting drug molecules, were also investigated. The sensor prototype and its feasibility for the detection of CFX are depicted in Scheme 1.

## 2. Experiments

### 2.1. Chemicals and Instruments

The reagents and chemicals utilised in the current experiment were of analytical grade. Potassium ferricyanide [K_3_Fe(CN)_6_], Copper chloride (CuCl_2_·2H_2_O), and potassium ferrocyanide [K_4_Fe(CN)_6_] were purchased from Himedia Pvt. Ltd. (Mumbai, India). Potassium chloride (KCl), sodium monophosphate (NaH_2_PO_4_), sodium bisphosphate (Na_2_HPO_4_), chloroauric acid (HAuCl_4_·3H_2_O), and cobalt (II) chloride were purchased from SRL Pvt. Ltd. (Mumbai, India). ddH_2_O from a Milli-Q water purifier was used to prepare standard solutions. Physical characterisation was carried out using an X-ray photoelectron spectroscope (XPS) (Central Instrument Facility Center (CIFC) IIT (BHU) Thermo Fisher K-Alpha instrument) and scanning electron microscope (SEM) (Central Instrument Facility Center (CIFC) IIT (BHU) Jeol, JCM-6000 Plus Bench Top Sem Neoscope) to examine the elemental compositions and the shape of the NDs at various magnifications. An electrochemical workstation (Palm Sense BV, Houten, The Netherlands) equipped with platinum wire (auxiliary electrode), GCE (the working electrode), and silver/silver chloride (Ag/AgCl) (the reference electrode) was used to evaluate the electrochemical nanoimprinting of the dendritic sensing probe and its analytical efficiency.

### 2.2. Preparation of GCE/AuCoCu Dendritic Sensing Probe

The electrode was first polished with alumina powder and then thoroughly rinsed with MilliQ water to wash away salts or ions. In order to electroimprint the trimetallic nanodendrites, AuCoCu over the GCE surface, 10 mM CuCl_2_·2H_2_O, 10 mM CoCl_2_·6H_2_O, and 10 mM HAuCl_4_·3H_2_O were dissolved in 0.1 M KCl solution. The potential and time of electrodeposition were optimised to check the nanodendritic growth. Further, control experiments were carried out by testing the trimetallic dendritic sensing probe, AuCoCu electrode, in a 0.5 M sulphuric acid (H_2_SO_4_) solution, 0.1 M sodium hydroxide (NaOH), and 0.1 M sodium sulphate (Na_2_SO_4_) in different experimental settings. This was conducted in order to verify the co-nanoimprinting and the presence of all three elements, Au, Co, and Cu on the same sensing surface. Monometallic Cu, Co, and Au dendritic sensing probes were also developed separately from 0.1 M KCl comprising 10 mM CoCl_2_·6H_2_O, 10 mM HAuCl_4_·3H_2_O, and 10 mM CuCl_2_·6H_2_O salts, respectively, for the comparative evaluation of all the electrodes surfaces. The surfaces were then washed with ethanol and distilled water and electrochemical measurements were carried out.

### 2.3. Real Sample Preparation

The trimetallic dendritic sensing probe was validated using the pharmaceutical formulation (capsules) and milk as the real sample. To analyse the effectiveness of AuCoCu for the detection of CFX, a conventional spike/recovery model was employed. The samples were equilibrated 10 times in phosphate-buffered saline (PBS) and then spiked with different concentrations of CFX. The current output was recorded and compared with the calibration plot obtained from a solution containing CFX in standard buffer.

### 2.4. Electrochemical Characterisations of the GCE/AuCoCu Dendritic Sensing Probe

The electrochemical characterisations of the trimetallic dendritic surface were performed in ferri/ferro cyanide solution (Zobbles solution (ZS)) (5 mM; pH-7) by using EIS, CV, and LSV. The EIS data were obtained at a ten points per decade sampling rate with an open circuit voltage ranging from 10 to 10^4^ Hz. Further amperometric responses were recorded between 0 and 60 s at a potential and t-equilibration of +1.2 V vs. Ag/AgCl and 10 s, respectively, to obtain the standard calibration plot of CFX.

## 3. Results and Discussions

### 3.1. Electrochemical Nanoimprinting of 3D AuCoCu Dendritic Sensing Probe

In order to verify the formation of the AuCoCu NDs, potentials of −0.6 V, −0.7 V, −0.8 V, and −0.9 V vs. Ag/AgCl were applied, where −0.8 V vs. Ag/AgCl showed the lowest resistance response in EIS and maximal capability to transmit electrons. Similarly, in order to optimise the time of electrodeposition, EIS was recorded at 10 s, 100 s, 300 s, 600 s, and 800 s. From 10 s to 600 s, the EIS showed a linear decline in resistance responses, which was most probably due to the increased branching and higher electron-transfer ability of AuCoCu NDs onto the electrode’s surface. However, at 800 s, resistance was higher in comparison with that at 600 s, the most probable reason being the insulation of the surface. The resulting trimetallic dendritic sensing probe was constructed using a simple and robust electroimprinting method (−0.8 V for 600 s), with a fabrication duration of less than 30 min. 

### 3.2. Physical Characterisation of 3D AuCoCu Dendritic Sensing Probe

**Figure 1:**
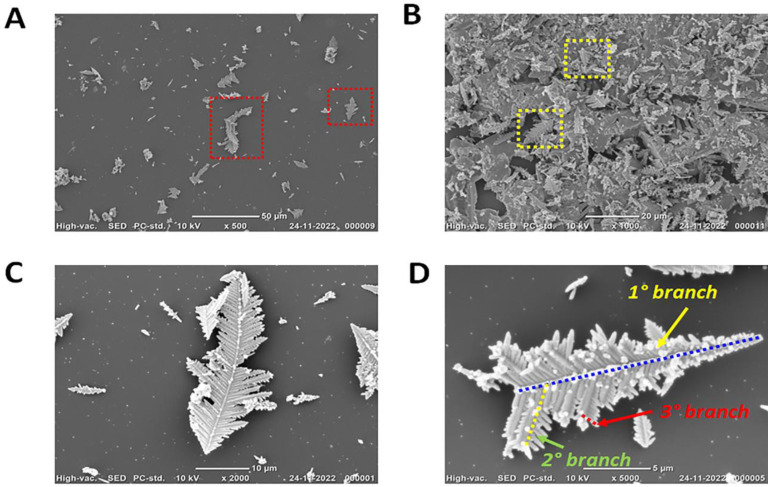
SEM images of imprinted trimetallic NDs at 50 µm, 20 µm, 10 µm, and 5 µm magnification, respectively (**A**–**D**).

**Figure 2:**
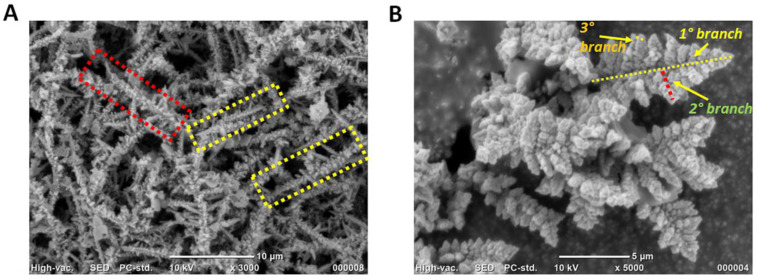
SEM images of imprinted trimetallic NDs at 10 µm and 5 µm magnification, respectively, on disposable SPCE (**A**,**B**).

In the first stage, the SEM was employed to examine the surface architecture of the formed trimetallic nanodendrites. The electrode was thoroughly cleaned and utilised for the SEM study to imprint the metallic dendrites. In order to achieve a homogenous dendritic pattern, the electrode was washed repeatedly and was electroimprinted; all other deposition-related parameters were identical to those listed in Section 3.1 above. Figure 1A–D show the well-grown dendritic hierarchical nanostructures over the surface at 50 µm, 20 µm, 10 µm, and 5 µm magnifications, respectively. The dotted box in (A–B) represents the dendritic imprint on the surface. The nanodendrites observed at various magnifications demonstrate the uniformity of materials formed by electroimprinting. The fern-like nanostructure’s primary (1°), secondary (2°), and tertiary (3°) branches were clearly observed in Figure 1D. The primary branch was significantly longer and measured 12 µm in length. Secondary branches, measuring between 3 and 8 µm, developed on the nodes of the parent branches and were significantly shorter and thinner. Tertiary branches were relatively smaller, measuring around 0.50 µm. In addition, the growth of trimetallic nanodendrites of AuCoCu was also tested on a screen-printed carbon electrode (SPCE) to determine whether such trimetallic nanoimprints can be deployable in field settings. Figure 2A,B show the imprinted trimetallic NDs at 10 µm and 5 µm magnification, respectively, on disposable SPCE. The dotted box in Figure 2A depicts the dendritic nanoimprint on the surface of SPCE, and the 1°, 2°, and 3° branches of the hierarchical nanostructure can be clearly observed in Figure 2B.

**Figure 3:**
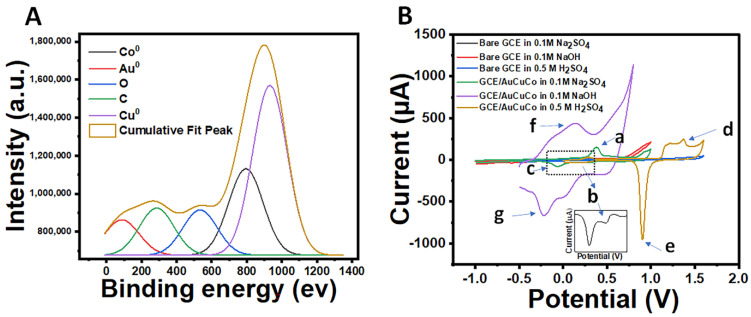
(**A**) XPS spectra of trimetallic NDs showing the elemental compositions in the imprinted system. (**B**) CV peak of GCE and GCE/AuCoCu dendritic surfaces in 0.1 M Na_2_SO_4_, 0.1 M NaOH, and 0.5 M H_2_SO_4_; the GCE/AuCoCu-imprinted surface showed a copper oxidation peak at +0.3 V (a) and reduction peaks at around +0.3 V (b) and −0.1 V (c) in 0.1 M Na_2_SO_4_ (green curve). Gold oxidation and reduction peaks (light brown curve) were obtained at approximately +1.3 V (d) and +0.9 V (e), respectively, in 0.5 M H_2_SO_4_. The cobalt peak showed oxidation and reduction peaks at +0.2 V (f) and −0.2 V (g) in 0.1 M NaOH (violet curve). These peaks verify the presence of copper (green curve), gold (light brown curve), and cobalt (violet curve) metals on the imprinted surface.

To further confirm the elemental compositions and the oxidation states of the various metals present on the surface, XPS measurements were carried out on the dendritic electroimprint generated at −0.8 V and 600 s. Figure 3A shows the XPS spectra of Cu2p, Au4f, and Co2p elements in trimetallic dendrites present on the surface. The imprinted dendrite produced peaks at around 932.0 eV, 84.00 eV, and 778.2 eV, pertaining to Cu^0^, Au^0^, and Co^0^, respectively, as shown in the figure, demonstrating that Cu, Au, and Co existed in their reduced metallic state. Further, the curve displayed an additional C 1s peak at around 284 eV and O 1s peak at around 536 eV. XPS analysis thus proved the presence of all three metals on the imprinted surface.

### 3.3. Validation of Au, Co, and Cu Trimetallic System Imprinted at the Electrode Surface

**Figure 4:**
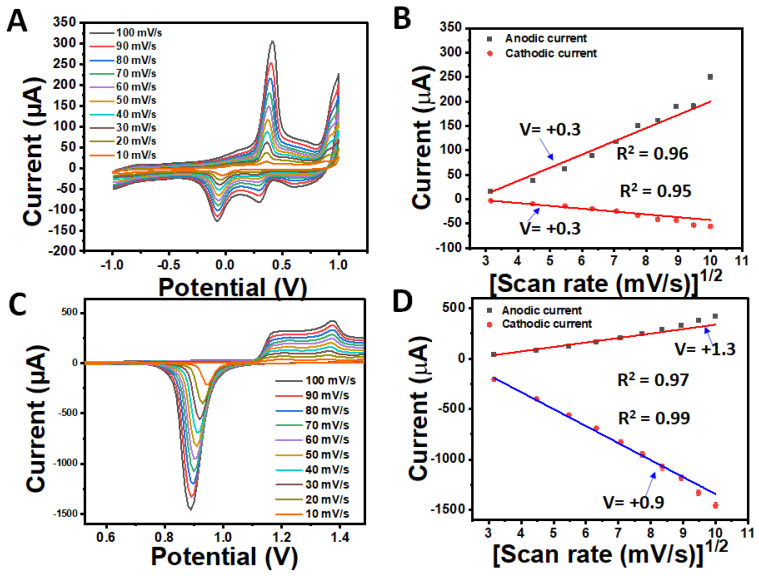
(**A**) Scan-rate-dependent experiment of GCE/AuCoCu-imprinted surface in 0.1 M Na_2_SO_4_ with the scan rate ranging from 10–100 mV/s; (**B**) Corresponding curve showing linearity, with a correlation coefficient of 0.96; (**C**) Scan-rate dependent experiment of the GCE/AuCoCu surface in 0.5 M H_2_SO_4_ with the scan rate ranging from 10–100 mV/s; (**D**) Corresponding current showing linearity, with a correlation coefficient of 0.97.

**Figure 5:**
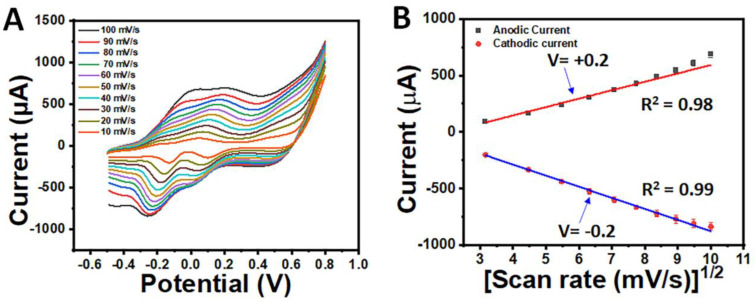
(**A**) Scan-rate-dependent experiment of GC/AuCoCu-imprinted surface in 0.1 M NaOH with the scan rate ranging from 10–100 mV/s; (**B**) Corresponding curve showing linearity, with a correlation coefficient of 0.98.

To further validate the presence of gold, cobalt, and copper on the surface of the electrode and electroimprinting, we conducted CV in 0.5 M H_2_SO_4_, 0.1 M NaOH, and 0.1 M Na_2_SO_4_ solutions in different experimental settings (Figure 3B). As gold, cobalt, and copper exhibited electrochemical performance under particular experimental settings, we chose distinct potential windows for these metals. Cobalt showed oxidation and reduction peaks at approximately +0.4 and −0.1 V vs. Ag/AgCl in 0.1 M NaOH solution, respectively, while copper showed an oxidation peak at +0.3 V vs. Ag/AgCl and reduction peaks at around +0.3 V and −0.1 V vs. Ag/AgCl in Na_2_SO_4_ solution. Gold displays an oxidation and reduction peaks at approximately +1.2 and +0.9 V vs. Ag/AgCl in 0.5 M H_2_SO_4_ solutions, respectively. First, we performed electroimprinting on the GCE surface at −0.8 V vs. Ag/AgCl for 600 s and repeatedly rinsed the electrodes with distilled water and ethanol. Then, we measured the CV response in 0.1 M Na_2_SO_4_ solution and found that copper oxidation was achieved at around +0.3 V vs. Ag/AgCl (a) and reduction peaks were obtained at +0.3 V vs. Ag/AgCl (b) and −0.1 V vs. Ag/AgCl (c) (green curve) [27]. The same electrode surface was repeatedly cleaned with ethanol and distilled water before being immersed in 0.5 M H_2_SO_4_ solution to verify the gold peak. And, as anticipated, we found gold oxidation and reduction peaks in CV at around +1.3 V vs. Ag/AgCl (d) and +0.9 V vs. Ag/AgCl (e), respectively (light brown curve) [28]. To confirm the cobalt peak, we dipped the electrode in 0.1 M NaOH and obtained oxidation and reduction peaks at +0.2 V vs. Ag/AgCl (f) and −0.2 V vs. Ag/AgCl (g) (violet curve), similar to those reported in the literature [29]. To confirm that the redox peaks recorded were simply caused by the presence of cobalt, gold, and copper metals in the sensing matrix, we again investigated the bare GCE in 0.5 M H_2_SO_4_, 0.1 M NaOH, and 0.1 M Na_2_SO_4_, respectively, under identical experimental circumstances. As there was no imprinting of a trimetallic system onto the GCE surface, no peaks were observed on bare GCE (blue, red, and black curves). Further, to validate the mechanism of electron transfer and stability of cobalt, gold, and copper, scan-rate-dependent analyses of the final dendritic sensing probe were carried out in 0.1 M NaOH, 0.5 M H_2_SO_4_, and 0.1 M Na_2_SO_4_ solutions, respectively. The cathodic (Ipc) and anodic (Ipa) peak currents were directly proportional to the square root of the scan rate, resulting in correlation coefficients of 0.98, 0.97, and 0.96 for cobalt, gold, and copper, respectively (Figure 4 and Figure 5). These findings evidently demonstrate that diffusion regulates electron transfer on the sensor’s surface and that the surface is highly stable, even at greater scan rates [30], making it an ideal device for pharmaceutical analysis and milk testing.

### 3.4. Electrochemical Characterisation of AuCoCu Dendritic Sensing Probe

**Figure 6:**
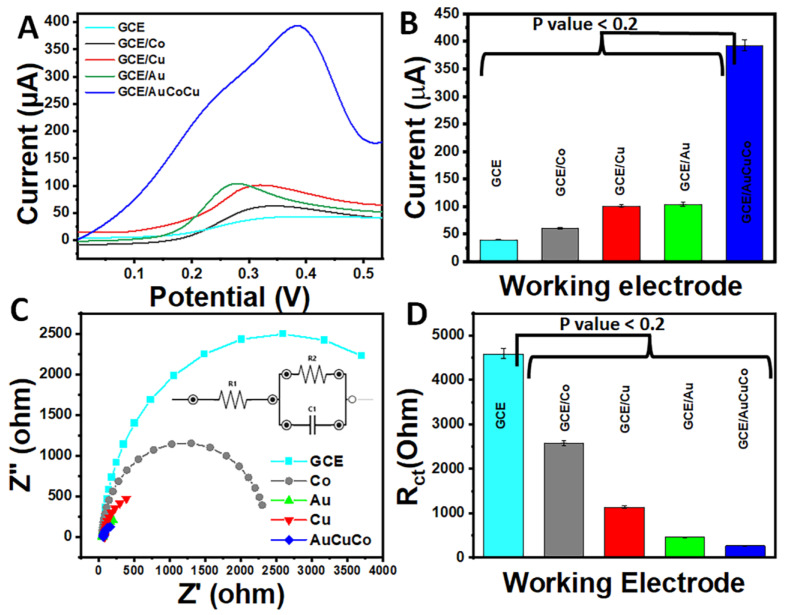
(**A**) LSV current signals of GCE (light blue curve), GCE/Co (grey curve), GCE/Cu (red curve), GCE/Au (green curve), and GCE/AuCoCu electrodes (blue curve) matrices in ZS (5 mM; pH-7, scan rate-100 mV/s); (**B**) Peak currents of electrode surfaces displayed in a comparative histogram; (**C**) EIS responses of GCE (light blue curve), GCE/Co (grey curve), GCE/Cu (red curve), GCE/Au (green curve), and GCE/AuCoCu electrode (blue curve) matrices with their circuit models; (**D**) Comparative histogram illustrating the corresponding Rct values.

After verifying the presence of all three metals on the sensing probe, the fabricated AuCoCu probe was electrochemically analysed to evaluate its electrocatalytic and conductive properties. We conducted LSV in 5 mM ZS at 50 mV/s scanning potential in a range between 0 and +0.4 V vs. Ag/AgCl (*n* = 5) (Figure 6A). A peak at approximately +0.3 V vs. Ag/AgCl was produced by the redox process of ZS at GCE (light blue curve), GCE/Co (grey curve), GCE/Cu (red curve), GCE/Au (green curve), and GCE/AuCoCu electrodes (blue curve), with an amplified peak of the GCE/AuCoCu electrodes. A comparison histogram displays the oxidative current response of each electrode’s surface (Figure 6B). The maximal current output in the case of GCE/AuCoCu may be attributed to the synergistic interactions of the Au, Co, and Cu components. It was statistically analysed, and the *p* value was <0.2. Furthermore, a minor shift in the anodic peak potential to the right was seen, indicating the trimetallic nanodendritic (AuCoCu) surface’s increased oxidation activity towards the ferri/ferro cyanide redox couple. This highlights that the imprinted nanodendritic surface was highly electrocatalytic in nature, which eventually improved the signal.

In order to quantify the charge transfer characteristics and electrocatalytic activities of the respective electrodes, we estimated the effective surface area (A) using Randles–Sevcik’s equation [31,32]

(1) $$I_{p}=\left(2.69\times 10^{5}\right)n^{3/2}\;ACD^{1/2}\;v^{1/2}\;$$

where *Ip* represents the highest current output (in amperes), *n* represents the number of electrons involved in the redox reaction (here, *n* = 1), *A* denotes the electrodes’ effective surface area (in cm^2^), *C* denotes the electroactive species’ concentration (in mole cm^−3^), *D* represents the diffusion coefficient (in cm^2^ s^−1^) with a value of 7.6 × 10^−6^ cm^2^s^−1^ for aqueous ferrocyanide, and *v* is the scan rate (in Vs^−1^).

The effective surface areas of GCE, GCE/Co, GCE/Cu, GCE/Au, and GCE/AuCoCu were calculated to be 0.047 cm^2^, 0.075 cm^2^, 0.124 cm^2^, 0.125 cm^2^, and 0.483 cm^2^, respectively. The value for the GCE/AuCoCu dendritic sensing probe was 10.27, 6.44, 3.89, and 3.86 times greater than those of bare GCE, GCE/Co, GCE/Cu, and GCE/Au, respectively. This proves that the imprinted dendritic sensing probe had a higher conductivity and could transfer electrons effectively.

To validate the LSV output, an EIS experiment was performed (Figure 6C) at the GCE (light blue curve), GCE/Co (grey curve), GCE/Cu (red curve), GCE/Au (green curve) and GCE/AuCoCu electrodes (blue curve) electrodes. The Rct’s values were 4589 (±0.52) Ω, 2580 (±0.38) Ω, 1135 (±0.62) Ω, 455.9 (±0.54) Ω, and 266 (±0.21) Ω, respectively. The EIS study supports the LSV findings, confirming that the GCE/AuCoCu NDs electrode had the lowest resistance or highest charge transfer kinetics (Figure 6D). This made it an ideal matrix for the electrochemical sensing of drug analysis.

**Figure 7:**
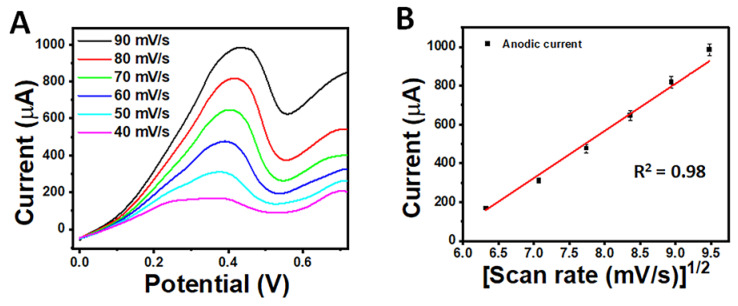
(**A**) Scan-rate-dependent experiment of the GCE/AuCoCu surface in ZS (5 mM; pH-7), with the scan rate ranging between 40 and 90 mV/s; (**B**) Corresponding current displaying linearity, with a correlation coefficient of 0.98.

To verify the stability and migration of ions to the GCE/AuCoCu dendritic sensing probe, LSV was monitored in 5 mM ZS solution at a scan rate between 40 and 90 mVs^−1^ (Figure 7A). The developed interface emphasised the sensor’s diffusion-controlled charge transfer behaviour and increased stability at higher potentials, as the scan rate’s square root was directly proportional to the cathodic (Ipc) and anodic (Ipa) peak currents, with a correlation coefficient of 0.98 (Figure 7B). The great charge-conducting capability of the imprinted probe signifies its potential for future applications.

### 3.5. Analytical Investigations of AuCoCu Nanoimprinted Dendrites

**Figure 8:**
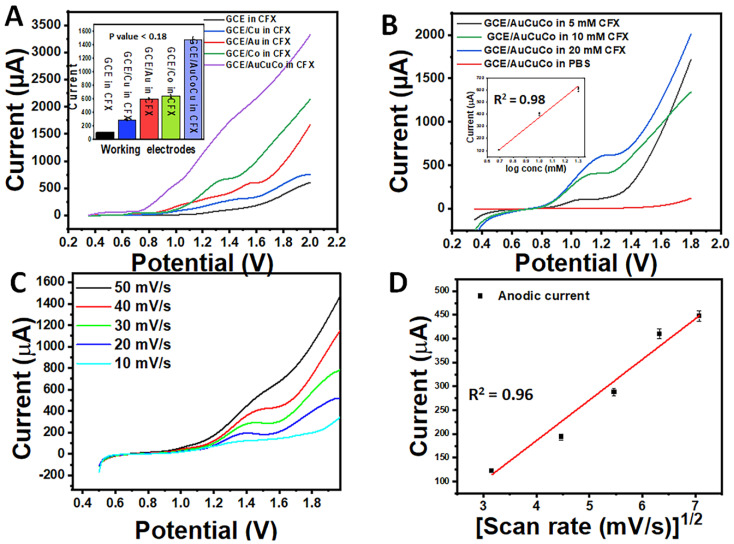
(**A**) LSV responses of GCE (black curve), GCE/Au (red curve), GCE/Co (green curve), GCE/Cu (blue curve), and GCE/AuCoCu (violet curve) matrices in 40 mM CFX dissolved in PBS (5 mM, pH 7, and scan rate 50 mV/s), inset showing the current histogram of all electrodes dipped in CFX; (**B**) Concentration-dependent control experiment of GCE/AuCoCu-imprinted matrix in PBS (red curve) and the GCE/AuCoCu surface in 5 mM (black curve), 10 mM (green curve), and 20 mM (blue curve) CFX, inset displaying the calibration plot; (**C**) Scan-rate-dependent study of GCE/AuCoCu matrix in 20 mM CFX, with the scan rate ranging between 10 and 50 mV/s; (**D**) Corresponding peak values showing a linear graph with a correlation coefficient of 0.96.

The efficacy of the electrochemically printed dendritic nanoprobe GCE/AuCoCu was analysed for sensing purposes by utilising CFX as a test molecule. To analyse the synergistic interactions of the trimetallic dendritic architecture in electroanalysis, we performed this study on all the electrode surfaces. First of all, LSV was conducted on bare GCE between +0.4 and +2.0 V vs. Ag/AgCl in PBS containing 40 mM CFX (*n* = 3) (Figure 8A). A low anodic peak (black curve) was observed at around +1.20 V vs Ag/AgCl as a result of the oxidation of CFX, which raised the electron density and, hence, the medium’s conductivity. When GCE/Au (red curve), GCE/Co (green curve), GCE/Cu (blue curve), and GCE/AuCoCu (violet curve) were examined under similar experimental settings, the oxidative signal outputs were enhanced, which was due to the prevalence of nanodendrites on the GCE’s surface. The baselines of all the matrices were similar, suggesting that the rise in the current magnitude was due to the electrochemical behaviour of CFX. Sulfoxide structures were produced as a result of oxidation occurring in the centre of the cephalosporin’s structure [14]. The fabricated sensing probe displayed an approximately 14-fold-greater current output than the bare GCE toward the oxidation of CFX because of the synergistic interactions amongst metal nanodendrites. A comparative histogram of all electrode surfaces for the oxidation of CFX was (inset of Figure 8A) statistically analysed, and the *p* value was <0.18. This emphasises the importance of the trimetallic AuCoCu surface in the sensing of CFX. In order to prove that the current at +1.2 V vs. Ag/AgCl was caused by the CFX’s direct transfer of electrons, control experiments were performed using the GCE/AuCoCu dendritic sensing probe. In the first control experiment, a concentration-dependent study of a dendritic sensing probe was performed in 5 mM PBS (blank) (red curve), and significantly higher concentrations of CFX in PBS, 5 mM (black curve), 10 mM (green curve), and 20 mM (blue curve). In the case of the blank, no peak was observed; however, the current outputs increased with increasing concentrations of CFX (Figure 8B). The linear curve showed a concentration-dependent signal, with a correlation coefficient of 0.98 (inset of Figure 8B). The regression equation obtained from the curve was as follows: ΔI (µA)  = −492.70 (± 47.5) + 868.00 (±65.67) × log Conc. [CFX (mM)].

In the second control test, we conducted a scan-rate-dependent study by altering the scan rate between 10 and 50 mV/s in the presence of 20 mM CFX (Figure 8C). As the scan rates increased, the anodic peak currents also increased and a linear curve was obtained, with a correlation coefficient of 0.96 (Figure 8D). Therefore, both control studies confirmed that the GCE/AuCoCu nano-imprinted probe was stable and could effectively sense CFX.

**Figure 9:**
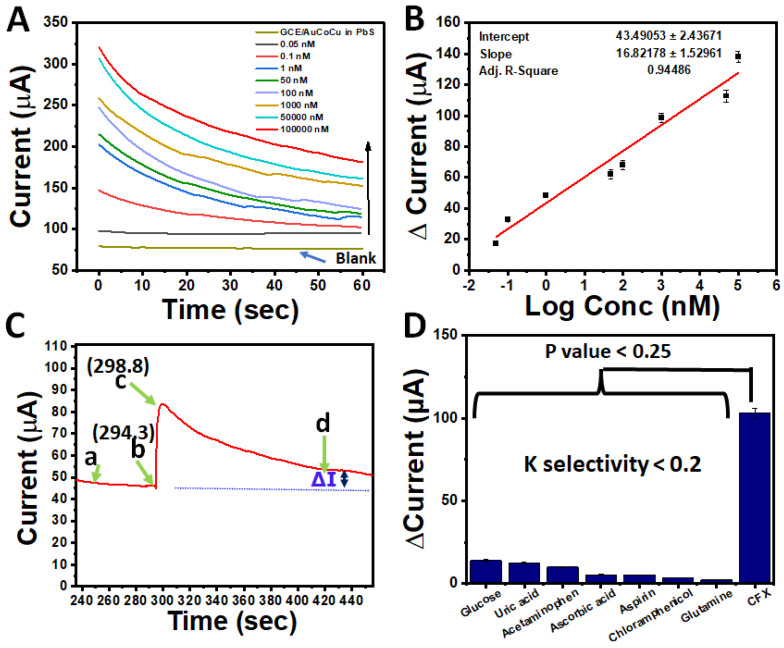
(**A**) Amperometric readout of GCE/AuCoCu-imprinted dendritic surface in the range of 0.05–10^5^ nm CFX in PBS; (**B**) Corresponding calibration curve of CFX in PBS; (**C**) Chronoamperometric profile of the GCE/AuCoCu dendritic sensing probe showing a quick response time of 4.5 ± 0.2 s; (a) represents the stable current; after injecting the CFX at point (b), a sharp spike in current (c) due to diffusion was recorded between 294.3 and 298.8 s; following that, current saturation occurred (d). (**D**) Selectivity of GCE/AuCoCu-imprinted dendritic surface towards several co-existing molecules and CFX.

The sensing capabilities of the GCE/AuCoCu nanoimprinted probe were further investigated using a very sensitive amperometric technique to find the minimal concentration, because there was a faint peak in the LSV. First of all, the signal output of the blank (without CFX) was recorded. In this case, a minimal peak at +1.2 V vs. Ag/AgCl was observed (light brown curve). The GCE/AuCoCu probe was evaluated for several concentrations of CFX in the following phase, and the signal output increased with an increase in the CFX levels (Figure 9A). Based on the amperometric readout, the calibration curve showed linearity in the concentration range of 0.05 nM to 10^5^ nM (Figure 9B). The LODs of CFX were calculated as 0.04 ± 0.01 nM using Equation (2)

*LOD* = 3 *σ_b_*/*m* (2)

where *σ_b_* represents the blank sample’s standard deviation and *m* is the calibration plot’s slope.

The linear increase in the amperometric responses with increasing CFX concentrations in the range of 0.05 to 10^5^ nM can be represented by the regression equation shown below: I (µA) = 43.49 (±2.43) + 16.82 (±1.5) log Conc. [CFX (nM)]. The response time, which is the length of time needed for the surface to exhibit a signal after analyte injection, had a substantial impact on the sensor’s efficiency. Chronoamperometry was carried out by injecting pharmaceutical capsules of cephalexin in blank PBS. We measured the current peak at +1.2 V vs. Ag/AgCl and obtained a response time of 4.5 ± 0.2 s. Figure 9C depicts the chronoamperometric curve after adding CFX to a stable current (a). After injecting the CFX at point (b), a sharp spike in current due to diffusion (c) was recorded between 294.3 and 298.8; after that, current saturation occurred (d). 

The produced 3D nanoimprinted dendritic sensing platform is appealing because it has a lower LOD, a faster response time, and can be constructed in 30 min without the need for cumbersome and conventional synthetic chemical processes. Additionally, we thoroughly evaluated the analytical performance of our sensor with recently published CFX sensors in Table 1. 

### 3.6. Selectivity Assay

The selectivity of the imprinted probe for a specific target in the presence of several existing molecules present in the complex matrix can be assessed to evaluate its commercial applications. Hence, we selected various co-existing molecules and drugs, namely glucose, uric acid, acetaminophen, ascorbic acid, aspirin, chloramphenicol, glutamine, and CFX, to examine the selectivity of the imprinted trimetallic dendritic sensing probe. The experiment was performed by recording the amperometric current signal in 50 µM concentrations at around +1.2 V vs. Ag/AgCl and the histogram in Figure 9D depicts the current signal of the co-existing molecule. The coefficient of selectivity (K_sel_) of the co-existing molecules was estimated using Equation (3), and the values are displayed in Table 2. It was significantly low (K_sel_ << 1), highlighting the good selectivity of the GCE/AuCoCu dendritic probe for CFX analysis.

*K_sel_* = (*Signal*)*_Interfering molecules_*/(*Signal*)*_cfx_* (3)

where *K_sel_* represents the coefficient of selectivity, (*Signal*)*_Interfering molecules_* is the current corresponding to interfering molecules, and (*Signal*)*_cfx_* is the response generated by the nanoprobe after being treated with *cfx*. 

The interfering molecules did not show any response in our experimental setup due to the CFX’s electrochemical passivity towards those molecules and/or the analytical voltage range, which prevented other electrochemically active species from undergoing the redox reaction. The statistical significance of the finding was assessed using the t-test, and the *p* value for the co-existing chemicals against CFX was found to be very low (<0.25, *n* = 5). Further, CFX was measured using the GCE/AuCoCu probe in pharmaceutical capsules and milk as a real sample in order to evaluate the feasibility of the sensing matrix.

### 3.7. Real Sample Analysis

CFX, an essential antibiotic of the cephalosporin family, is used to treat a variety of infections. However, its use has led to the emergence of resistant strains of microorganisms, which have a negative impact on a person’s health; hence, rapid sensing of CFX is of clinical interest. A GCE/AuCoCu sensing matrix was challenged for its ability to detect CFX in commercially available phexin pharmaceutical capsules. We conducted spike and recovery tests using pharmaceutical capsules to evaluate the potential of the constructed dendritic sensing probes for real samples. First, capsule powder was equilibrated ten times in PBS, and it was then spiked with various quantities of CFX. The current signal was then recorded under the optimised experimental conditions. Using Equation (4), the recovery percentage of CFX at different concentrations was calculated.

% *Recovery* = ([*S*]*_cfx_* − [*B*]*_cfx_*)/[*SS*]*_cfx_* × 100 (4)

where [*S*]*_cfx_* and [*B*]*_cfx_* are the analytical responses of the GCE/AuCoCu nanoimprinted dendrites in real samples with cephalexin spiked and blank samples, respectively, and [*SS*]*_cfx_* represents the response in the standard buffer with the same concentration of *cfx*. 

**Figure 10:**
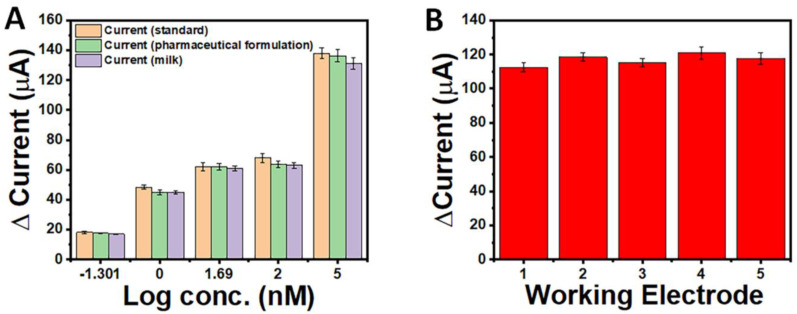
(**A**) Real sample assay depicting a higher percentage recovery of CFX in the pharmaceutical formulations and milk sample in the range of 0.05 nm–10^5^ nm; (**B**) Histogram showing the chip-to-chip reproducibility of the three-dimensional nanoimprinted dendritic surface.

In Figure 10A, the green histograms represent the current output for varied concentrations of CFX in pharmaceutical samples (*n* = 3). The current increased linearly as the CFX concentration increased, and the current recoveries for different CFX concentrations ranged between 93.29 and 99.77% (RSD < 3.5%) when compared with the standard assay (orange histogram). The linear increase in the signal output with increasing CFX concentrations can be mathematically depicted as follows: ΔI (µA) = 39.11 (±2.21) + 16.49 (±1.56) log Conc. [CFX (nM)]. The sensor efficacy was very high in pharmaceutical formulations, with a correlation coefficient of 0.96. During the analysis, it was found that the peak current was marginally lower than the standard. The causes of these variations could be handling errors and restrictions of ion mobility in real sample matrices. The pharmaceutical formulation consisted of a variety of components; however, they did not interfere with our experiment and had no adverse effects on the sensing matrix. 

CFX drugs are frequently used to treat animals raised for food production. However, these medications and their metabolites can often enter the food chain and endanger human health [33]. In order to evaluate the applicability of the trimetallic-imprinted surface, we also tested for CFX in milk samples. Various concentrations of CFX were spiked in milk samples and their current signals were recorded under the optimised experimental conditions. In Figure 10A, violet histograms represent the current output for varied concentrations of CFX in milk samples (*n* = 3). With an increase in the CFX concentration, the peak current increased, and recoveries were found to be between 92.66 and 98.29% (RSD < 3.5%) when compared with the standard assay (orange histogram). The linear increase in the signal output with increasing CFX concentrations can be mathematically depicted as follows: ΔI (µA) = 38.83 (±2.32) + 16.25 (±1.75) log Conc. [CFX (nM)], with a correlation coefficient of 0.95. These outcomes clearly indicate that the proposed electrochemically printed trimetallic sensor is robust and can precisely sense CFX in diverse biological matrices at various orders of magnitude, revealing its enormous commercial prospects.

### 3.8. Reproducibility and Stability Assay

Five different AuCoCu ND electrodes were separately tested to analyse the chip-to-chip reproducibility. Sensing electrodes were constructed in various batches, and the current response was measured for CFX. The variation in the chip-to-chip current response was very low, with an RSD of <4.5% (Figure 10B). The reason for this minor change in the output current could be a handling error or a variation in the other test conditions. We also examined a single dendritic nanoprobe’s capability to analyse CFX five times in succession without any pre-treatment, and the RSD was <3.7%. Due to the excellent stability of the nanodendritic architecture and the absence of any biorecognition elements (enzymes, antibodies, etc.) during the fabrication of the sensor, the GCE/AuCoCu NDs sensing matrix had good reproducibility The long-term stability of the nanomaterial-imprinted sensing probe was also periodically examined for 8 weeks. The current was recorded at 1-week intervals, and it was observed that 95–97% of the CFX signal was retained up to 6 weeks (RSD < 3.2%). Hence, the results demonstrate that the imprinted nanomaterial is a reliable sensing matrix with up to 6 weeks of stability and has high reproducibility, which is essential for its preservation and shelf life.

## 4. Conclusions

We developed an electrochemically nanoimprinted 3D AuCoCu nanodendritic surface. The printed surface was precisely utilised for the direct sensing of CFX. The matrix was constructed by optimising the electrodeposition variables for superior bioelectronics performance. The GCE/AuCoCu NDs sensing surface was characterised using XPS, SEM, and several electrochemical techniques, including chronoamperometry, LSV, and EIS. The calibration curve showed linearity in the concentration range between 0.05 nM and 10^5^ nM, with a limit of detection of 0.04 ± 0.01 nM. The dendritic sensing probe had a response time of 4.5 ± 0.2 s, highlighting its excellent sensitivity to CFX determination. When evaluated in the presence of significant concentrations of both electroinactive and electroactive interfering molecules that are common in biological samples, the sensor showed high CFX selectivity, with K_sel_ << 1. The constructed sensor accurately sensed CFX in a real matrix, and the outcomes were consistent with the standard calibration curve. The suggested sensor exhibited good percentage recoveries and appears to be a promising platform for sensing CFX in pharmaceutical formulations and milk. To the best of our knowledge, this is the first report of AuCoCu trimetallic dendritic nanostructures created by a rapid one-step electroimprinting process with superior conductive and catalytic properties. In future, the nanoimprinted dendritic surface could be integrated with a multiplexing device and can be used for the simultaneous detection of analytes. Such an integrated approach, in our opinion, will be essential for pharmacological analysis and could even be advantageous for overall human health. 

## Figures and Tables

**Table 1:** Comparative analysis of nanoimprinted AuCoCu dendrites with the recently reported CFX sensors (NR).

S. No.	Electrode Fabrication	LDR	LOD	Response Time	Real Sample	Reference
1	BDDE/MIP	10–1000 nM	4.9 nM	NR	River water and pharmaceutical samples	[9]
2	Sensor chip/CM/Dextran/Cephalexin	0.667–10.689 nm	-	NR	Milk sample	[33]
3	MIP-coated silica nanoparticle	50,000–6 × 10^5^ nM	1600 nM	NR	Urine	[34]
4	Carbon nanodots	500–9500 nM	311 nM	6 min	NR	[3]
5	Poly(resorcinol)/GCE	100–3 × 10^5^ nM	3.12 nM	NR	Blood, urine, And tablet samples	[35]
6	Bare BDDE	500–7 × 10^5^ nM,	95 nM	NR	Capsules, river water, and human urine	[14]
7	**GCE/AuCoCu NDs**	**0.05–1 × 10^5^ nM**	**0.04 ± 0.01 nM**	**4.5 ± 0.2 s**	**Pharmaceutical capsules and milk**	**This work**

**Table 2:** Various interfering molecules with coefficients of selectivity (*K_sel_*).

Co-Existing Molecules	Current Signal	K_sel_
Glucose	14	0.14
Uric acid	12.23	0.11
Acetaminophen	10.04	0.10
Ascorbic acid	5.35	0.05
Aspirin	5.20	0.05
Chloramphenicol	3.4	0.03
Glutamine	2.30	0.02
CFX	102.9	1

## Data Availability

Not applicable.

## References

[1] Herman T.F., Hashmi M.F. (2022). Cephalexin.

[2] Lai E.P.C., Wu S.G. (2003). Molecularly Imprinted Solid Phase Extraction for Rapid Screening of Cephalexin in Human Plasma and Serum. Anal. Chim. Acta.

[3] Wang K., Guan F., Li H., Li M., Feng H., Fan H. (2015). One-Step Synthesis of Carbon Nanodots for Sensitive Detection of Cephalexin. RSC Adv..

[4] Benarab N., Fangninou F.F. (2020). The Issues of Antibiotics: Cephalexin Antibiotic as Emerging Environment Contaminant. Int. J. Sci. Res. Publ..

[5] DaCunha M., Moore S., Kaplan D. (2018). Cephalexin-Induced Acute Generalized Exanthematous Pustulosis. Dermatol. Rep..

[6] Lan L., Yao Y., Ping J., Ying Y. (2017). Recent Advances in Nanomaterial-Based Biosensors for Antibiotics Detection. Biosens. Bioelectron..

[7] Zhou C., Zou H., Sun C., Li Y. (2021). Recent Advances in Biosensors for Antibiotic Detection: Selectivity and Signal Amplification with Nanomaterials. Food Chem..

[8] Stevenson H.S., Shetty S.S., Thomas N.J., Dhamu V.N., Bhide A., Prasad S. (2019). Ultrasensitive and Rapid-Response Sensor for the Electrochemical Detection of Antibiotic Residues within Meat Samples. ACS Omega.

[9] Feier B., Blidar A., Pusta A., Carciuc P., Cristea C. (2019). Electrochemical Sensor Based on Molecularly Imprinted Polymer for the Detection of Cefalexin. Biosensors.

[10] Percin-Ozkorucuklu S., Uka B., Yildirim-Bastemur G. (2019). Voltammetric Analysis of Cephalexin and Cefazolin in Pharmaceutical Formulations and Biological Samples. J. Turk. Chem. Soc. Sect. A Chem..

[11] Won S.Y., Chandra P., Hee T.S., Shim Y.B. (2013). Simultaneous Detection of Antibacterial Sulfonamides in a Microfluidic Device with Amperometry. Biosens. Bioelectron..

[12] Duanghathaipornsuk S., Farrell E.J., Alba-Rubio A.C., Zelenay P., Kim D.S. (2021). Detection Technologies for Reactive Oxygen Species: Fluorescence and Electrochemical Methods and Their Applications. Biosensors.

[13] Lobnik A., Turel M., Urek Š.K., Wen W. (2012). Optical Chemical Sensors: Design and Applications. Advances in Chemical Sensors.

[14] Feier B., Gui A., Cristea C., Săndulescu R. (2017). Electrochemical Determination of Cephalosporins Using a Bare Boron-Doped Diamond Electrode. Anal. Chim. Acta.

[15] Saxena V., Chandra P., Pandey L.M. (2018). Design and Characterization of Novel Al-Doped ZnO Nanoassembly as an Effective Nanoantibiotic. Appl. Nanosci..

[16] Akhtar M.H., Hussain K.K., Gurudatt N.G., Chandra P., Shim Y.B. (2018). Ultrasensitive Dual Probe Immunosensor for the Monitoring of Nicotine Induced-Brain Derived Neurotrophic Factor Released from Cancer Cells. Biosens. Bioelectron..

[17] Mahato K., Prasad A., Maurya P., Chandra P. (2016). Nanobiosensors: Next Generation Point-of-Care Biomedical Devices for Personalized Diagnosis. J. Anal. Bioanal. Technol..

[18] Chandra P., Prakash R. (2020). Nanobiomaterial Engineering: Concepts and Their Applications in Biomedicine and Diagnostics.

[19] Kumari R., Dkhar D.S., Mahapatra S., Divya, Singh S.P., Chandra P. (2022). Nano-Engineered Surface Comprising Metallic Dendrites for Biomolecular Analysis in Clinical Perspective. Biosensors.

[20] Purohit B., Kumar A., Mahato K., Chandra P. (2020). Electrodeposition of Metallic Nanostructures for Biosensing Applications in Health Care. J. Sci. Res..

[21] Purohit B., Kumar A., Mahato K., Srivastava A., Chandra P. (2022). Engineered Three-Dimensional Au-Cu Bimetallic Dendritic Nanosensor for Ultrasensitive Drug Detection in Urine Samples and in Vitro Human Embryonic Kidney Cells Model. Microchem. J..

[22] Oladipo A.O., Nkambule T.T.I., Mamba B.B., Msagati T.A.M. (2020). Therapeutic Nanodendrites: Current Applications and Prospects. Nanoscale Adv..

[23] Khumngern S., Choosang J., Thavarungkul P., Kanatharana P., Numnuam A. (2020). Flow Injection Enzyme-Free Amperometric Uric Acid Sensor Consisting of Ordered Mesoporous Carbon Decorated with 3D Pd-Pt Alloy Nanodendrite Modified Screen-Printed Carbon Electrode. Microchem. J..

[24] Purohit B., Kumar A., Mahato K., Chandra P. (2020). Novel Sensing Assembly Comprising Engineered Gold Dendrites and MWCNT-AuNPs Nanohybrid for Acetaminophen Detection in Human Urine. Electroanalysis.

[25] Lv H., Li Y., Zhang X., Gao Z., Feng J., Wang P., Dong Y. (2018). The Label-Free Immunosensor Based on Rhodium@palladium Nanodendrites/Sulfo Group Functionalized Multi-Walled Carbon Nanotubes for the Sensitive Analysis of Carcino Embryonic Antigen. Anal. Chim. Acta.

[26] Ge S., Zhang Y., Zhang L., Liang L., Liu H., Yan M., Huang J., Yu J. (2015). Ultrasensitive Electrochemical Cancer Cells Sensor Based on Trimetallic Dendritic Au@PtPd Nanoparticles for Signal Amplification on Lab-on-Paper Device. Sens. Actuators B Chem..

[27] Protich Z., Santhanam K.S.V., Jaikumar A., Kandlikar S.G., Wong P. (2016). Electrochemical Deposition of Copper in Graphene Quantum Dot Bath: Pool Boiling Enhancement. J. Electrochem. Soc..

[28] Ma W., Ying Y.L., Qin L.X., Gu Z., Zhou H., Li D.W., Sutherland T.C., Chen H.Y., Long Y.T. (2013). Investigating Electron-Transfer Processes Using a Biomimetic Hybrid Bilayer Membrane System. Nat. Protoc..

[29] Zheng J.Y., Quan Z.L., Song G., Kim C.W., Cha H.G., Kim T.W., Shin W., Lee K.J., Jung M.H., Kang Y.S. (2012). Vertical Cobalt Dendrite Array Films: Electrochemical Deposition and Characterization, Glucose Oxidation and Magnetic Properties. J. Mater. Chem..

[30] Allen J., Bard Larry R. (2019). Faulkner ELECTROCHEMICAL METHODS Fundamentals and Applications Allen.

[31] Rodríguez Presa M.J., Gassa L.M., Azzaroni O., Gervasi C.A. (2009). Estimating Diffusion Coefficients of Probe Molecules into Polyelectrolyte Brushes by Electrochemical Impedance Spectroscopy. Anal. Chem..

[32] Wang L., Bai J., Huang P., Wang H., Zhang L., Zhao Y. (2006). Self-Assembly of Gold Nanoparticles for the Voltammetric Sensing of Epinephrine. Electrochem. Commun..

[33] Dillon P.P., Daly S.J., Browne J.G., Manning B.M., Loomans E., Van Amerongen A., O’Kennedy R. (2003). Application of an Immunosensor for the Detection of the β-Lactam Antibiotic, Cephalexin. Food Agric. Immunol..

[34] Sahu S., Karuppusamy M., Easwaramoorthi S. (2022). Water-Dispersible Polymer Coated Silica Nanoparticle for Turn-on Fluorometric Detection of Cephalexin. Biosens. Bioelectron. X.

[35] Kassa A., Amare M., Benor A., Tigineh G.T., Beyene Y., Tefera M., Abebe A. (2022). Potentiodynamic Poly(Resorcinol)-Modified Glassy Carbon Electrode as a Voltammetric Sensor for Determining Cephalexin and Cefadroxil Simultaneously in Pharmaceutical Formulation and Biological Fluid Samples. ACS Omega.

